# A Class of Nonlocal Coupled Semilinear Parabolic System with Nonlocal Boundaries

**DOI:** 10.1155/2014/912356

**Published:** 2014-02-16

**Authors:** Hong Liu, Haihua Lu

**Affiliations:** ^1^Teaching Group of Mathematics, Zhenjiang Watercraft College of PLA, Zhenjiang 212003, China; ^2^School of Science, Nantong University, Nantong 226007, China

## Abstract

We investigate the positive solutions of the semilinear parabolic system with coupled nonlinear nonlocal sources subject to weighted nonlocal Dirichlet boundary conditions. The blow-up and global existence criteria are obtained.

## 1. Introduction

In this paper, we consider the positive solutions of the semilinear parabolic system with coupled nonlinear nonlocal sources subject to weighted nonlocal Dirichlet boundary conditions:

(1) $$\begin{array}{c} u_{it}=\Delta u_{i}+\int_{\Omega }u_{i}^{q_{i}}u_{i+1}^{p_{i}}\left(x,t\right)\text{d}x,\\ i=1,2,\ldots ,k,\;u_{k+1}=u_{1},\;x\in \Omega ,\;t>0,\\ u_{i}\left(x,t\right)=\int_{\Omega }\varphi_{i}\left(x,y\right)u_{i}\left(y,t\right)\text{d}y,\\ i=1,2,\ldots ,k,\;x\in \partial \Omega ,\;t>0,\\ u_{i}\left(x,0\right)=u_{i,0}\left(x\right),\;i=1,2,\ldots ,k,\;\;x\in \Omega , \end{array}$$

where *Ω* is a bounded domain in ℝ^*N*^, *N* ≥ 1, with smooth boundary ∂*Ω*. The exponents *p*_*i*_ > 0, *q*_*i*_ ≥ 0. The weighted functions *φ*_*i*_ in the boundary conditions are continuous, nonnegative on $\partial \Omega \times \bar{\Omega }$ and ∫_*Ω*_ *φ*_*i*_(*x*, *y*)d*y* > 0 on ∂*Ω*. The initial data $u_{i0}(x)\in C^{2+\nu }(\bar{\Omega })$ with 0 < *ν* < 1, *u*_*i*0_(*x*) ≥ 0, ≢0, and satisfy the compatibility conditions.

Many physical phenomena were formulated into nonlocal mathematical models and studied by many authors [1–13]. For example, in [1], Bebernes and Bressan studied an ignition model for a compressible reactive gas which is a nonlocal reaction-diffusion equation. Furthermore, Bebernes et al. [14] considered a more general model:

(2) $$\begin{array}{c} u_{t}-\Delta u=f\left(u\right)+g\left(t\right),\;x\in \Omega ,\;\;t>0,\\ u\left(x,0\right)=u_{0}\left(x\right),\;x\in \Omega ,\\ u\left(x,t\right)=0,\;x\in \partial \Omega ,\;\;t>0, \end{array}$$

where *u*_0_(*x*) ≥ 0, *g*(*t*) > 0 or *g*(*t*) = (*k*/|*Ω*|)∫_*Ω*_ *u*_*t*_(*x*, *t*) d *x* with *k* > 0. Chadam et al. [15] studied another form of (2) with *f*(*u*) = 0 and *g*(*t*) = ∫_*Ω*_ *ψ*(*u*(*x*, *t*))d*x* and proved that the blow-up set is the whole region (including the homogeneous Neumann boundary conditions). Souplet [16, 17] considered (2) with the general function *g*(*t*). Pao [18] discussed a nonlocal reaction-diffusion equation arising from the combustion theory.

The problems with both nonlocal sources and nonlocal boundary conditions have been studied as well. To motivate our study, we give a short review of examples of such parabolic equations or systems studied in the literature. For example, Lin and Liu [19] studied the following problem:

(3) $$\begin{array}{c} u_{t}-\Delta u=\int_{\Omega }f\left(u\left(y,t\right)\right)\text{d}y,\;x\in \Omega ,\;\;t>0,\\ u\left(x,t\right)=\int_{\Omega }\varphi \left(x,y\right)u\left(y,t\right)\text{d}y,\;x\in \partial \Omega ,\;\;t>0,\\ u\left(x,0\right)=u_{0}\left(x\right),\;x\in \Omega ; \end{array}$$

they established local existence, global existence, and nonexistence of solutions and discussed the blow-up properties of solutions.

Gladkov and Kim [20] considered the problem of the form

(4) $$\begin{array}{c} u_{t}=\Delta u+c\left(x,t\right)u^{p},\;x\in \Omega ,\;\;t>0,\\ u\left(x,t\right)=\int_{\Omega }\varphi \left(x,y\right)u^{l}\left(y,t\right)\text{d}y,\;x\in \partial \Omega ,\;\;t>0,\\ u\left(x,0\right)=u_{0}\left(x\right),\;x\in \Omega , \end{array}$$

with *p*, *l* > 0. And some criteria for the existence of global solution as well as for the solution to blow up in finite time were obtained.

In [21], Kong and Wang studied system (1) when *k* = 2:

(5) ut=Δu+∫Ωum(x,t)vn(x,t)dx, x∈Ω,  t>0,vt=Δv+∫Ωup(x,t)vq(x,t)dx, x∈Ω,  t>0,u(x,t)=∫Ωφ(x,y)u(y,t)dy,v(x,t)=∫Ωψ(x,y)v(y,t)dy,x∈∂Ω,  t>0,u(x,0)=u0(x), v(x,0)=v0(x), x∈Ω;

they obtained the following results, and we extend them as follows.

(i) Assume that *m*, *q* < 1 and *np* ≤ (1 − *m*)(1 − *q*) hold; then the solution of (5) exists globally.
(ii) If one of the following conditions holds:
  (6) (a)  m>1,(b)  q>1,(c)  np>(1−m)(1−q),
  then the solution of (5) blows up in a finite time for the sufficiently large initial data.
(iii) Assume that ∫_*Ω*_ *φ*(*x*, *y*)d*y* ≥ 1 and ∫_*Ω*_ *ψ*(*x*, *y*)d*y* ≥ 1 for all *x* ∈ ∂*Ω* and one of (6) holds; then the solution of problem (5) blows up in a finite time for any positive initial data.

Recently, Zheng and Kong [22] also studied the following problem:

(7) $$\begin{array}{c} u_{t}-\Delta u=u^{m}\left(x,t\right)\int_{\Omega }v^{n}\left(x,t\right)\text{d}x,\;x\in \Omega ,\;\;t>0,\\ v_{t}-\Delta v=v^{q}\left(x,t\right)\int_{\Omega }u^{p}\left(x,t\right)\text{d}x,\;x\in \Omega ,\;\;t>0, \end{array}$$

with the same initial and boundary conditions as (5), and they established similar conditions for global and nonglobal solutions and also blow-up solutions.

The main purpose of this paper is to get the blow-up criterion of problem (1) for any positive integer *k*.

In the following, we set *Q*_*T*_ = *Ω* × (0, *T*), and  *S*_*T*_ = ∂*Ω* × (0, *T*) with 0 < *T* < *∞* for convenience.

It is known by the standard theory [16, 23] that there exists a local positive solution to (1). Moreover, by the comparison principle (see Lemma 10 in the next section), the uniqueness of solutions holds if *p*_*i*_, *q*_*i*_ ≥ 1, *i* = 1,2,…, *k*.

**Theorem 1**

Problem (1) has a positive classical solution $(u_{1},u_{2},\ldots ,u_{k})\in {\left[C^{2+\hat{\alpha },1+\hat{\alpha }/2}(Q_{T})\cap C({\bar{Q}}_{T})\right]}^{k}$ for some $\hat{\alpha }:0<\hat{\alpha }<1$. Moreover, if *T* < *∞*, then

(8) $$\begin{array}{c} \underset{t\to T}{\lim}\left({||u_{1}\left(\cdot ,t\right)||}_{\infty }+\cdots +{||u_{k}(\cdot ,t)||}_{\infty }\right)=\infty . \end{array}$$

**Theorem 2**

If exponents *p*_*i*_, *q*_*i*_, *i* = 1,2,…, *k* satisfy

(9) $$\begin{array}{c} q_{i}<1,\;i=1,2,\ldots ,k,\\ p_{1}p_{2}\cdots p_{k}\leq \left(1-q_{1}\right)\left(1-q_{2}\right)\cdots \left(1-q_{k}\right), \end{array}$$

the solution (*u*_1_, *u*_2_,…, *u*_*k*_) of (1) exists globally for any nontrivial nonnegative initial data.

**Theorem 3**

If exponents *p*_*i*_, *q*_*i*_, *i* = 1,2,…, *k* satisfy one of the following:

(10) (a)  qr>1, r∈{1,2,…,k},(b)  p1p2⋯pk>(1−q1)(1−q2)⋯(1−qk)

and if ∫_*Ω*_ *φ*_*i*_(*x*, *y*) d *y* < 1, *i* = 1,2,…, *k*, for all *x* ∈ ∂*Ω*, then the solution of (1) exists globally for small nonnegative initial data.

**Theorem 4**

If exponents *p*_*i*_, *q*_*i*_, *i* = 1,2,…, *k* satisfy one of the following:

(11) (a)  qr>1, r∈{1,2,…,k},(b)  p1p2⋯pk>(1−q1)(1−q2)⋯(1−qk),

then the solution of (1) blows up in finite time for large initial data.

If the initial data *u*_*i*,0_(*x*) satisfies

(12) $$\begin{array}{c} \left(H\right)\;\Delta u_{i,0}+\int_{\Omega }^{}u_{i,0}^{q_{i}}u_{i,0+1}^{q_{i}}\geq 0,\;i=1,2,\ldots ,k, \end{array}$$

we have another blow-up result.

**Theorem 5**

Assume that

(13) $$\begin{array}{c} \;\;q_{r}>1,\;\;\int_{\Omega }\varphi_{r}\left(x,y\right)\text{ d }y\geq 1,\;r\in \left\{1,2,\ldots ,k\right\} \end{array}$$

and the condition (*H*) holds. Then the solution of (1) blows up in finite time for any positive initial data.

This paper is organized as follows.  Section 2 is devoted to some comparison principles. In Section 3, we prove two global existence results. The blow-up results are proved in the final section.

## 2. Comparison Principle

Before proving the main results, we give the maximum and comparison principles related to the problem. First, we give the following definition of the upper and lower solutions.

**Definition 6**

A pair of functions $\left({\bar{u}}_{1}(x,t),\ldots ,{\bar{u}}_{k}(x,t)\right)$ is called an upper solution of (1), if, for every *i* = 1,2,…, *k*,  ${\bar{u}}_{i}(x,t)\in C^{2,1}(Q_{T})\cap C({\bar{Q}}_{T})$ and satisfies

(14) u¯it≥Δu¯i+∫Ωu¯iqiu¯i+1pi(x,t)dx,  u¯k+1=u¯1,x∈Ω,  t>0,u¯i(x,t)≥∫Ωφi(x,y)u¯i(y,t)dy, x∈∂Ω,  t>0,u¯i(x,0)≥ui,0(x), x∈Ω.

Similarly, a lower solution of (1) is defined by the opposite inequalities.

**Lemma 7**

Suppose that $a_{ij},b_{i},f_{i}\in C({\bar{Q}}_{T})$ and *f*_*i*_ ≥ 0,  *c*_*i*_, *d*_*i*_ ≥ 0 in *Q*_*T*_, *g*_*i*_(*x*, *y*) ≥ 0 on $\partial \Omega \times \bar{\Omega }$, ∫_*Ω*_ *g*_*i*_(*x*, *y*) d *y* > 0 on ∂*Ω*, *i* = 1,2,…, *k*, *j* = 1,2,…, *N*. If, for every *i* = 1,2,…, *k*, $w_{i}\in C^{2,1}(Q_{T})\cap C({\bar{Q}}_{T})$ and satisfies

(15) wit−Δwi≥∑j=1Naij∂wi∂xj+biwi+fi(x,t)∫Ω(ciwi+diwi+1)
d
x, (x,t)∈QT,wi(x,t)≥∫Ωgi(x,y)wi(y,t)
d
y, (x,t)∈ST,wi(x,0)>0, x∈Ω,

where *w*_*k*+1_ = *w*_1_, then *w*_*i*_(*x*, *t*) > 0, *i* = 1,2, on ${\bar{Q}}_{T}$.

**Proof**

Set ${\bar{b}}_{i}=\sup_{{\bar{Q}}_{T}}|b_{i}|$, *z*_*i*_ = *e* ^−*Kt*^ *w*_*i*_ with $K>\max\{{\bar{b}}_{i},i=1,2,\ldots ,k\}$. Then

(16) zit−Δzi+(K−bi)zi ≥∑j=1Naij∂zi∂xj+fi(x,t)∫Ω(cizi+dizi+1)dx, (x,t)∈QT,zi(x,t)≥∫Ωgi(x,y)zi(y,t)dy, (x,t)∈ST,zi(x,0)>0, x∈Ω.

Since *z*_*i*_(*x*, 0) > 0, *i* = 1,2,…, there exists *δ* > 0 such that *z*_*i*_ > 0 for $\left(x,t)\in \bar{\Omega }\times (0,\delta \right)$. Suppose for a contradiction that $\bar{t}=\sup\{t\in (0,T):z_{i}>0\;\mathrm{on}\;\bar{\Omega }\times [0,t),i=1,2,\ldots ,k\}<T$. Then *z*_*i*_ ≥ 0 on ${\bar{Q}}_{\bar{t}}$, and at least one of *z*_*i*_ vanishes at $\left(\bar{x},\bar{t}\right)$ for some $\bar{x}\in \bar{\Omega }$. Without loss of generality, suppose that $z_{1}(\bar{x},\bar{t})=0=\inf_{{\bar{Q}}_{\bar{t}}}z_{1}$. If $(\bar{x},\bar{t})\in Q_{\bar{t}}$; by virtue of the first inequality of (16), we find that

(17) $$\begin{array}{c} z_{1t}-\Delta z_{1}+\left(K-b_{1}\right)z_{1}-\sum_{j=1}^{N}a_{ij}\frac{\partial z_{i}}{\partial x_{j}}\geq 0,\;\left(x,t\right)\in {\bar{Q}}_{\bar{t}}. \end{array}$$

This leads to the conclusion that *z*_1_ ≡ 0 in ${\bar{Q}}_{\bar{t}}$ by the strong maximum principle, a contradiction. If $(\bar{x},\bar{t})\in S_{\bar{t}}$, this results in a contradiction too, that

(18) $$\begin{array}{c} 0=z_{1}\left(\bar{x},\bar{t}\right)=\int_{\Omega }g_{1}\left(x,y\right)z_{1}\left(y,t\right)\text{d}y>0 \end{array}$$

due to ∫_*Ω*_ *g*_1_(*x*, *y*)d*y* > 0 on ∂*Ω*. This proves that *z*_1_ > 0 and consequently *w*_1_ > 0. We complete the proof.

**Lemma 8**

Suppose that, for every *i* = 1,2,…, *k*, $w_{i}\in C^{2,1}(Q_{T})\cap C({\bar{Q}}_{T})$ and satisfies

(19) $$\begin{array}{c} w_{it}-\Delta w_{i}\geq \int_{\Omega }\left(a_{i}\left(x,t\right)w_{i}+b_{i}\left(x,t\right)w_{i+1}\right)\text{ d }x,\;\left(x,t\right)\in Q_{T},\\ w_{i}\left(x,t\right)\geq \int_{\Omega }g_{i}\left(x,y\right)w_{i}\left(y,t\right)\text{ d }y,\;\left(x,t\right)\in S_{T},\\ w_{i}\left(x,0\right)\geq 0,\;x\in \Omega , \end{array}$$

where *w*_*k*+1_ = *w*_1_ and *a*_*i*_(*x*, *t*), *b*_*i*_(*x*, *t*) are continuous, nonnegative functions in ${\bar{Q}}_{T}$, *g*_*i*_(*x*, *y*) ≥ 0 on $\partial \Omega \times \bar{\Omega }$ such that ∫_*Ω*_ *g*_*i*_(*x*, *y*) d *y* < 1 on ∂*Ω*, and there exist positive constants *C*_*i*_ such that ∫_*Ω*_(*a*_*i*_(*x*, *t*) + *b*_*i*_(*x*, *t*)) d *x* ≤ *C*_*i*_. Then *w*_*i*_(*x*, *t*) ≥ 0, *i* = 1,2, on ${\bar{Q}}_{T}$.

**Proof**

Suppose that the strict inequalities of (19) hold; by Lemma 7, we have *w*_*i*_(*x*, *t*) > 0. Now we consider the general case. Set

(20) $$\begin{array}{c} v_{i}=w_{i}+ɛe^{Kt}, \end{array}$$

where *ɛ* is any fixed positive constant, and *K* = 1 + max⁡{∫_*Ω*_(*a*_*i*_(*x*, *t*) + *b*_*i*_(*x*, *t*)) d *x*, *i* = 1,2,…, *k*}. By (19), we get, for *i* = 1,2,…, *k*,

(21) vit−Δvi−∫Ω(ai(x,t)vi+bi(x,t)vi+1)dx ≥ɛeKt(K−∫Ω(ai(x,t)+bi(x,t))dx)>0, (x,t)∈QT,vi(x,t)−∫Ωgi(x,y)vi(y,t)dy ≥ɛeKt(1−∫Ωgi(x,y)dy)>0, (x,t)∈ST,vi(x,0)≥ɛeKt>0, x∈Ω,

Therefore, we have *v*_*i*_(*x*, *t*) ≥ 0 on *Q*_*T*_. Letting *ɛ* → 0^+^, we get the desired result.

If the boundary condition ∫_*Ω*_ *g*_*i*_(*x*, *y*)d*y* < 1 is not necessarily valid, we have the following result. The argument of its proof can be referred to [22, Lemma 2.2].

**Lemma 9**

Suppose that $a_{ij},b_{i},f_{i}\in C({\bar{Q}}_{T})$, *f*_*i*_ ≥ 0, *c*_*i*_, *d*_*i*_, are nonnegative and bounded in *Q*_*T*_, *g*_*i*_(*x*, *y*) ≥ 0 on $\partial \Omega \times \bar{\Omega }$, ∫_*Ω*_ *g*_*i*_(*x*, *y*) d *y* > 0 on ∂*Ω*, *i* = 1,2,…, *k*, *j* = 1,2,…, *N*. If, for every *i* = 1,2,…, *k*,  $w_{i}\in C^{2,1}(Q_{T})\cap C({\bar{Q}}_{T})$ and satisfies

(22) wit−Δwi≥∑j=1Naij∂wi∂xj+biwi+fi(x,t)∫Ω(ciwi+diwi+1)
d
x, (x,t)∈QT,wi(x,t)≥∫Ωgi(x,y)wi(y,t)
d
y, (x,t)∈ST,wi(x,0)≥0, x∈Ω,

where *w*_*k*+1_ = *w*_1_, then *w*_*i*_(*x*, *t*) ≥ 0, *i* = 1,2, on ${\bar{Q}}_{T}$.

By Lemma 9, we can easily get the following result.

**Lemma 10**

Let $\left({\bar{u}}_{1},{\bar{u}}_{2},\ldots ,{\bar{u}}_{k}\right)$ and $\left({\underline{u}}_{1},{\underline{u}}_{2},\ldots ,{\underline{u}}_{k}\right)$ be nonnegative upper and lower solution of system (1) on ${\bar{Q}}_{t}$, respectively. If one assumes that, for some *r* ∈ {1,2,…, *k*},

- ${\bar{u}}_{r+1}>\delta$ or ${\underline{u}}_{r+1}>\delta$ when *p*_*r*_ < 1,
- ${\bar{u}}_{r}>\delta$ or ${\underline{u}}_{r}>\delta$ when *q*_*r*_ < 1,

then $\left({\bar{u}}_{1},{\bar{u}}_{2},\ldots ,{\bar{u}}_{k})\geq ({\underline{u}}_{1},{\underline{u}}_{2},\ldots ,{\underline{u}}_{k}\right)$ on *Q*_*T*_.

## 3. Global Existence Results

Before proving Theorem 2, we give a global existence result for a scalar equation.

**Lemma 11**

Let *w*_0_(*x*) and *φ*(*x*, *y*) be continuous, nonnegative functions on $\bar{\Omega }$ and $\partial \Omega \times \bar{\Omega }$, respectively, and let the nonnegative constants *θ*_*ij*_ satisfy 0 < *θ*_*i*1_ + *θ*_*i*2_ ≤ 1. Then the solutions of the nonlocal problem

(23) $$\begin{array}{c} w_{t}-\Delta w=\sum_{i=1}^{k}w^{\theta_{i1}}\left(x,t\right)\int_{\Omega }w^{\theta_{i2}}\left(x,t\right)\text{ d }x,\;x\in \Omega ,\;\;t>0,\\ w\left(x,t\right)=\int_{\Omega }\varphi \left(x,y\right)w\left(y,t\right)\text{ d }y,\;x\in \partial \Omega ,\;\;t>0,\\ w\left(x,0\right)=w_{0}\left(x\right),\;x\in \Omega \end{array}$$

exist globally.

**Proof**

The augment is similar to the proof of [22, Lemma 3.1] or [21, Lemma 6]. For the reader's convenience, we complete it. It is easy to prove that there exists a positive function $\psi \in C^{2}(\bar{\Omega })$ such that

(24) min⁡Ω¯⁡ ψ(x)>max⁡Ω¯⁡ w02(x),ψ(x)≥∫Ωφ2(x,y)dy∫Ωψ(y)dy,x∈∂Ω.

Let *θ* > 0 be large enough such that

(25) 2θ min⁡Ω¯⁡ ψ(x) ≥(2k+1)max⁡{max⁡Ω¯⁡|Δψ(x)|,|Ω|[max⁡Ω¯ ψ(x)](θi1+θi2+1)/2(i=1,2,…,k)|Ω|}.

Setting *z*(*x*, *t*) = *e* ^2*θt*^ *ψ*(*x*) for (*x*, *t*) ∈ *Ω* × (0, *∞*), one readily checks that

(26) zt−Δz≥2∑i=1kz(θi1+1)/2(x,t)∫Ωzθi2/2(x,t)dx,x∈Ω,  t>0,z(x,t)≥∫Ωφ2(x,y)dy∫Ωz(y,t)dy, x∈∂Ω,  t>0,z(x,0)≥w02(x)+1, x∈Ω,

Let $\bar{w}=z^{1/2}(x,t)$; it follows that

(27) $$\begin{array}{c} {\bar{w}}_{t}-\Delta \bar{w}\geq \sum_{i=1}^{k}{\bar{w}}^{\theta_{i1}}\left(x,t\right)\int_{\Omega }{\bar{w}}^{\theta_{i2}}\left(x,t\right)\text{d}x,\;x\in \Omega ,\;\;t>0,\\ \bar{w}\left(x,t\right)\geq \int_{\Omega }\varphi^{2}\left(x,y\right)\text{d}y\int_{\Omega }\bar{w}\left(y,t\right)\text{d}y,\;x\in \partial \Omega ,\;\;t>0,\\ \bar{w}\left(x,0\right)>w_{0}\left(x\right),\;x\in \Omega . \end{array}$$

This implies that $\bar{w}$ is a global upper solution of (23). Clearly, 0 is a lower solution of it. So we complete the proof.

**Proof of Theorem 2**

By (11), we know that there exists *a*_*i*_ ∈ (0,1), *i* = 1,2,…, *k*, such that

(28) $$\begin{array}{c} \frac{p_{i}}{1-q_{i}}\leq \frac{a_{i}}{a_{i+1}},\;i=1,2,\ldots ,k,\;\;a_{k+1}=a_{1}. \end{array}$$

Define *α* = ∑_*i*=1_^*k*^1/*a*_*i*_. Let Φ(*x*, *y*) ≥ max⁡{*φ*_*i*_(*x*, *y*), *i* = 1,2,…, *k*} be a continuous function defined for $(x,y)\in \partial \Omega \times \bar{\Omega }$. Suppose that *z* solves

(29) $$\begin{array}{c} z_{t}-\Delta z=\alpha \sum_{i=1}^{k}z^{1-a_{i}}\left(x,t\right)\int_{\Omega }z^{a_{i}}\left(x,t\right)\text{d}x,\;x\in \Omega ,\;\;t>0,\\ z\left(x,t\right)=\sum_{i=1}^{k}g_{i}\left(x\right)\int_{\Omega }\Phi \left(x,y\right)z\left(y,t\right)\text{d}y,\;x\in \partial \Omega ,\;\;t>0,\\ z\left(x,0\right)=1+\sum_{i=1}^{k}u_{i,0}^{1/a_{i}}\left(x\right),\;x\in \Omega , \end{array}$$

where

(30) $$\begin{array}{c} g_{i}\left(x\right)={\left(\int_{\Omega }\Phi \left(x,y\right)\text{d}y\right)}^{\left(1-a_{i}\right)/a_{i}}. \end{array}$$

In view of Lemma 11, we know that *z* is global. Moreover, *z* > 1 in $\bar{\Omega }\times [0,\infty )$ by the maximum principle. Set ${\bar{u}}_{i}=z^{a_{i}}$, *i* = 1,2,…, *k*. By (28) and (29) and using H$\ddot{\text{o}}$lder's inequality, we get

(31) u−it−Δu−i−∫Ωu−iqiu−i+1pidx =aizai−1zt−aizai−1Δz−ai(ai−1)|∇z|2  −∫Ωzaiqi+ai+1pidx ≥aizai−1(zt−Δz)−∫Ωzaidx≥(αai−1)∫Ωzaidx ≥0, (x,t)∈QT,u−i−∫Ωφi(x,y)u−i(y,t)dy =zai−∫Ωφi(x,y)zai(y,t)dy ≥(∫Ωφi(x,y)dy)1−ai(∫Ωφi(x,y)z(y,t)dy)ai  −∫Ωφi(x,y)zai(y,t)dy ≥0, (x,t)∈ST,u−i(x,0)≥ui,0(x), x∈Ω.

This means that $\left({\bar{u}}_{1},{\bar{u}}_{2},\ldots ,{\bar{u}}_{k}\right)$ is a global upper solution of (1).

**Proof of Theorem 3**

Define

(32) $$\begin{array}{c} \max\left\{\underset{\partial \Omega }{\sup}\int_{\Omega }\varphi_{i}\left(x,y\right)\text{d}y,i=1,2,\ldots ,k\right\}=\delta_{0}\in \left(0,1\right). \end{array}$$

Let *w* be the unique solution of the elliptic problem

(33) $$\begin{array}{c} -\Delta w=1,\;x\in \Omega ;\;w=C_{0},\;\;x\in \partial \Omega . \end{array}$$

Then there exists a constant *M* > 0 such that *C*_0_ ≤ *w*(*x*) ≤ *C*_0_ + *M* in $\bar{\Omega }$. We choose *C*_0_ to be large enough such that

(34) $$\begin{array}{c} \frac{1+C_{0}}{1+C_{0}+M}\geq \delta_{0}. \end{array}$$

Set ${\bar{u}}_{i}(x,t)=b_{i}(1+w(x))$. When (*x*, *t*) ∈ *S*_*T*_, it follows that

(35) u¯i−∫Ωφi(x,y)u¯i(y,t)dy=bi(1+C0)−bi∫Ωφi(x,y)(1+w(y))dy≥bi[1+C0−(1+C0+M)δ0]≥0.

Now we investigate (*x*, *t*) ∈ *Q*_*T*_. Set *L*_*i*_ = (1 + *C*_0_ + *M*)^*p*_*i*_+*q*_*i*_^ | *Ω*| for convenience. A simple computation yields

(36) u¯it−Δu¯i−∫Ωu¯iqiu¯i+1pidx =bi−biqibi+1pi∫Ω(1+w(x))pi+qidx ≥biqi(bi1−qi−bi+1piLi).

(a) If *q*_*r*_ > 1, no matter *q*_*r*+1_ > 1 or *q*_*r*+1_ ≤ 1, we can choose *b*_*r*_ to be small enough such that *b*_*r*_^1−*q*_*r*_^ ≥ *b*_*r*+1_^*p*_*r*_^ *L*_*r*_. For fixed *b*_*r*_, there exist *b*_*i*_, *i* = 1,2,…, *r* − 1, *r* + 1,…, *k*, satisfying *b*_*i*_^1−*q*_*i*_^ ≥ *b*_*i*+1_^*p*_*i*_^ *L*_*i*_, *i* = 1,2,…, *k*. It follows that

(37) $$\begin{array}{c} {\bar{u}}_{it}-\Delta {\bar{u}}_{i}-\int_{\Omega }{\bar{u}}_{i}^{q_{i}}{\bar{u}}_{i+1}^{p_{i}}\text{d}x\geq 0,\;i=1,2,\ldots ,k. \end{array}$$

(b) If *q*_*i*_ ≤ 1, *i* = 1,2,…, *k* and *p*_1_ *p*_2_ ⋯ *p*_*k*_ > (1 − *q*_1_)(1 − *q*_2_)⋯(1 − *q*_*k*_), we can choose *b*_1_ to be small enough such that

(38) b1(1−q1)(1−q2)⋯(1−qk) >b1p1p2⋯pkL1(1−q2)⋯(1−qk)L2p1(1−q3)⋯(1−qk)  ⋯Lk−1p1p2⋯pk−2(1−qk−1)Lkp1p2⋯pk−1.

Consequently, there exist *b*_*i*_ > 0, *i* = 2,3,…, *k*, *b*_*k*+1_ = *b*_1_ satisfying *b*_*i*_^1−*q*_*i*_^ ≥ *b*_*i*+1_^*p*_*i*_^ *L*_*i*_, *i* = 1,2,…, *k*. Hence (37) holds too.

By (35) and (37), in any case (a) or (b), we know that the solution of (1) must be global for small data *u*_*i*,0_(*x*) ≤ *b*_*i*_(1 + *w*(*x*)), *i* = 1,2,…, *k* for *x* ∈ *Ω*.

## 4. Blow-Up Results

In this section, we assume that (*u*(*x*, *t*), *v*(*x*, *t*)) is a positive solution of (1) on $\bar{\Omega }\times [0,T)$, where *T* is the maximal existence time.

**Proof of Theorem 4**

We denote by *λ*_1_, *ϕ*_1_(*x*) the first eigenvalue and the corresponding eigenfunction of the linear elliptic problem:

(39) $$\begin{array}{c} -\Delta \varphi \left(x\right)=\lambda \varphi \left(x\right),\;x\in \Omega ;\;\;\varphi \left(x\right)=0,\;x\in \partial \Omega , \end{array}$$

and *ϕ*_1_(*x*) satisfies

(40) $$\begin{array}{c} \varphi_{1}\left(x\right)>0,\;x\in \Omega ,\;\underset{\bar{\Omega }}{\max}{\;\phi }_{1}\left(x\right)=1. \end{array}$$

Define *γ* = min⁡{*α*_*i*_(*q*_*i*_ − 1) + *α*_*i*+1_ *p*_*i*_ + 1, *i* = 1,2,…, *k*}.

(a) If *q*_*r*_ ≥ 1, we claim that there exist positive constants *α*_*i*_ > 1, *i* = 1,2,…, *k*, such that the inequality

(41) $$\begin{array}{c} \alpha_{i}\left(q_{i}-1\right)+\alpha_{i+1}p_{i}>0 \end{array}$$

holds. First, when *i* = *r*, (41) holds for any *α*_*r*_, *α*_*r*+1_ > 1. When *i* = *r* + 1, if *q*_*r*+1_ ≥ 1, (41) holds for any *α*_*r*+2_ > 1; if *q*_*r*+1_ ≤ 1 we can choose *α*_*r*+2_ > max⁡{1, *α*_*r*+1_(1 − *q*_*r*+1_)/*p*_*r*+1_}. That is, (41) holds too. When *i* = *r* − 1, if *q*_*r*−1_ ≥ 1, (41) holds for any *α*_*r*−1_ > 1; if *q*_*r*−1_ < 1, we can choose 1 < *α*_*r*−1_ < (*α*_*i*_ *p*_*r*−1_/1 − *q*_*r*−1_) such that (41) holds too.

(b) If *q*_*i*_ < 1, *i* = 1,2,…, *k*, and *p*_1_ *p*_2_ ⋯ *p*_*k*_ > (1 − *q*_1_)(1 − *q*_2_) ⋯ (1 − *q*_*k*_), we can choose *α*_*i*_ > 1 such that

(42) $$\begin{array}{c} \frac{p_{1}}{1-q_{1}}>\frac{\alpha_{1}}{\alpha_{2}},\;\;\frac{p_{2}}{1-q_{2}}>\frac{\alpha_{2}}{\alpha_{3}},\ldots ,\frac{p_{k}}{1-q_{k}}>\frac{\alpha_{k}}{\alpha_{1}}. \end{array}$$

Hence (41) holds too.

Hence, for the case (a) or (b), we all have *γ* > 1. Now let *s*(*t*) be the unique solution of the ODE problem

(43) $$\begin{array}{c} s^{\prime }\left(t\right)=-\lambda s\left(t\right)+ls^{\gamma }\left(t\right),\;t>0,\\ s\left(0\right)=s_{0}>1, \end{array}$$

where *l* = min⁡{(1/*α*_*i*_)∫_*Ω*_ *ϕ*_1_^*α*_*i*_*q*_*i*_+*α*_*i*+1_*p*_*i*_^, *i* = 1,2,…, *k*}. Then *s*(*t*) blows up in finite time *T*(*s*_0_) with *s*_0_ being large enough.

Set

(44) u_i=sαi(t)ϕ1αi(x), (x,t)∈Ω¯×[0,T(s0)),i=1,2,…,k.

We will show that $\left(\underline{u},\underline{v}\right)$ is a lower solution of problem (1). A direct computation yields

(45) u_it−Δu_i−∫Ωu_iqiu_i+1pidx =αilsαi−1+γϕ1αi−αi(αi−1)sαiϕ1αi−2|∇ϕ1|2  −∫Ωsαiqi+ai+1piϕ1αiqi+ai+1pidx ≤αilsαi−1+γ−sαiqi+ai+1pi∫Ωϕ1αiqi+ai+1pidx ≤0, (x,t)∈Ω−×[0,T(s0)),u_i−∫Ωφi(x,y)u_i(y,t)dy =0−sαi(t)∫Ωφi(x,y)ϕ1αi(y)dy ≤0, (x,t)∈∂Ω×(0,T(s0)).

$\left({\underline{u}}_{1},\ldots ,\underline{u_{k}}\right)$ is a blowing up lower solution of (1) provided the initial data are so large that *u*_*i*,0_(*x*) ≥ *s* ^*α*_*i*_^(0)*ϕ*_1_^*α*_*i*_^(*x*), *i* = 1,2,…, *k* for *x* ∈ *Ω*. We complete the proof.

**Proof of Theorem 5**

Since *u*_*i*,0_ > 0 in *Ω*, ∫_*Ω*_ *φ*_*r*_(*x*, *y*) d *y* > 0 on ∂*Ω*, and

(46) $$\begin{array}{c} u_{i,0}\left(x\right)=\int_{\Omega }\varphi_{r}\left(x,y\right)u_{i,0}\left(y\right)\text{ d }y,\;x\in \partial \Omega , \end{array}$$

by the compatibility conditions, we have *u*_*i*,0_ > 0 on ∂*Ω*. Denote by *η* the positive constant such that *u*_*i*,0_ > *η* on $\bar{\Omega }$. The assumption (*H*) implies that (*u*_*i*_)_*t*_ > 0 by the comparison principle, and in turn *u*_*i*_ > *η*, *i* = 1,2,…, *k* on $\bar{\Omega }\times [0,T)$. Furthermore, *u*_*r*_ satisfies

(47) $$\begin{array}{c} {\left(u_{r}\right)}_{t}\geq \Delta u_{r}+\left|\Omega \right|\eta^{p_{r}}u_{r}^{q_{r}},\;\left(x,t\right)\in Q_{T},\\ u_{r}=\int_{\Omega }\varphi_{r}\left(x,y\right)u_{r}\left(y,t\right)\text{ d }y,\;\left(x,t\right)\in S_{T},\\ u_{r}\left(x,0\right)=u_{r,0}\left(x\right),\;x\in \Omega . \end{array}$$

Let *z*_*r*_(*t*) be the solution of the following Cauchy problem:

(48) $$\begin{array}{c} z_{r}^{\prime }\left(t\right)=\left|\Omega \right|\eta^{p_{r}}z_{r}^{q_{r}},\\ z_{r}\left(0\right)=\frac{1}{2}\eta >0. \end{array}$$

Clearly, *z*_*r*_(*t*) blows up under the condition

(49) $$\begin{array}{c} q_{r}>1. \end{array}$$

On the other hand, since ∫_*Ω*_ *φ*_*r*_(*x*, *y*) d *y* ≥ 1, by Lemma 9, we have *u*_*r*_ ≥ *z*_*r*_ as long as both *u*_*r*_ and *z*_*r*_ exist, and thus *u*_*r*_ blows up for any positive initial data. The proof now is completed.
